# Clinical utility of plasma microbial cell-free DNA sequencing for early diagnosis of toxoplasmosis in high-risk patients: a five-patient case series

**DOI:** 10.1017/ash.2026.10382

**Published:** 2026-05-11

**Authors:** Fernando H. Centeno, Todd Lasco, Mayar Al Mohajer

**Affiliations:** 1 Baylor College of Medicine, USA; 2 University of Oxford, Oxford, UK

## Abstract

From 2017 to 2025, we reviewed plasma microbial cell-free DNA sequencing (mcfDNA NGS) results detecting Toxoplasma gondii. Five patients were identified. Median turnaround was 3 days (2–5). mcfDNA initiated therapy in two and supported empiric treatment in three but required clinical context for interpretation.

## Introduction

Toxoplasmosis, caused by *Toxoplasma gondii*, usually persists as lifelong latent infection. Reactivation or donor-derived infection can cause severe disease in immunocompromised hosts, especially people with advanced Human Immunodeficiency Virus (HIV) infection and solid-organ transplant recipients.^1–3^ Central Nervous System (CNS) disease is common in HIV, whereas disseminated disease can occur in transplant recipients and other immunocompromised hosts.

Diagnosis of CNS toxoplasmosis is often presumptive. In HIV, neuroimaging classically shows multiple ring-enhancing lesions, but the differential includes primary CNS lymphoma and other opportunistic infections.^2^ Serology documents exposure but cannot distinguish latent infection from reactivation; after transplantation, early infection may occur before seroconversion.^3^ Cerebrospinal fluid (CSF) *T. gondii* polymerase chain reaction (PCR) is highly specific but has modest sensitivity, highest before or within the first week of therapy, and lumbar puncture or brain biopsy may be delayed or contraindicated in critically ill patients.^2,4^

Plasma microbial cell-free DNA next-generation sequencing (mcfDNA NGS) is an unbiased assay that detects and quantifies microbial DNA fragments in plasma and can identify a broad range of pathogens without targeted primers.^5^ In multicenter studies, plasma mcfDNA testing has been associated with changes in antimicrobial management, but interpretation requires diagnostic stewardship because co-detection of organisms and uncertain negative predictive value are common.^6,7^ Evidence specific to toxoplasmosis is limited to case reports and small series.^8,9^ We describe a single-center five-patient case series of plasma mcfDNA NGS identifying *T. gondii* and compare turnaround time (TAT) and clinical impact with conventional testing.

## Methods

We performed a retrospective case series of patients with plasma mcfDNA NGS (Karius, Redwood City, CA) positive for *T. gondii* at Baylor St. Luke’s Medical Center (Houston, Texas) from January 1, 2017, through December 31, 2025.

A result was considered positive when *T. gondii* was reported by the assay; quantitative concentrations (molecules per microliter [MPM]) were recorded when provided. The assay’s analytical validation supports a reporting threshold of >10 MPM.^5^

We abstracted demographics, immunocompromising conditions, presenting syndrome, imaging findings, diagnostic testing, treatment decisions, and outcomes from the electronic medical record. Clinical TAT was defined as the time from test order entry to result availability.

Toxoplasma IgG testing was performed in-house; IgM testing was sent to a reference laboratory (Quest Diagnostics, San Juan Capistrano, CA); and *T. gondii* PCR testing (when performed) was sent to the University of Washington (Seattle, WA).

This study was reviewed and approved by the Baylor College of Medicine Institutional Review Board (protocol H-45143) with a waiver of informed consent for de-identified retrospective analysis.

## Results

Five patients were identified (median age, 48 yr; range, 39–67); four were male. Three had HIV/AIDS (CD4 range, 3–10^6^ cells/mm^3^), one was a heart transplant recipient (donor seropositive/recipient seronegative), and one was immunocompetent. Three presented with new neurologic symptoms and ring-enhancing brain lesions, one had neurologic symptoms without compatible lesions, and one had prolonged febrile systemic illness with hepatitis after travel. Patient-level findings are summarized in Table 1.

**Table 1.** Clinical characteristics and diagnostic testing among patients with plasma mcfDNA NGS positive for *Toxoplasma gondii* (2017–2025)

Patient	Toxoplasma PCR	Toxoplasma serology, serum (TAT)	Cultures and pathology	mcfDNA NGS organisms detected (MPM when available) and clinical TAT	Change in management	Disposition and outcome
49-yr-old male with recently diagnosed HIV/AIDS on ART presenting with headaches and vomiting and found to have a new basal ganglia mass.	Cerebrospinal fluid PCR: positive (7 days)	IgM: negative (3 days) IgG: positive (2 days)	Bone marrow pathology: negative (5 days) Brain biopsy: Cerebral toxoplasmosis by immunoperoxidase stains (1 day)	*Toxoplasma gondii*: not quantified Enterococcus faecium: not quantified Enterococcus faecalis: not quantified Nocardia farcinica: not quantified Cytomegalovirus: not quantified (2 days)	No, already on empiric trimethoprim-sulfamethoxazole	Discharged to long-term acute-care hospital on pyrimethamine/leucovorin and clindamycin with corticosteroid taper
67-yr-old male with epilepsy and a non-ischemic cardiomyopathy and an orthotopic heart transplant (donor Toxoplasma positive, recipient negative) 14 months prior presenting with chronic fatigue, dyspnea, unstable gait, and breakthrough seizures.	Cerebrospinal fluid PCR: negative (3 days)	IgM: positive (5 days) IgG: negative (2 days)	None	Torque teno virus 6 (1860) *Toxoplasma gondii* (955) Varicella-zoster virus (241) *Staphylococcus epidermidis* (131) *Rothia mucillaginosa* (97) Cytomegalovirus (86) *Lactobacillus gasseri* (85) (2 days)	Yes, started on trimethoprim-sulfamethoxazole	Discharged to long-term acute care hospital on trimethoprim-sulfamethoxazole
45-yr-old female returning from travel in Peru with fever, a rash, influenza-like symptoms, and transaminase elevation	None	IgM: positive (9 days) IgG: positive (2 days)	None	*Toxoplasma gondii* (9227) *Klebsiella pneumoniae* (4273) (3 days)	Yes, started on trimethoprim-sulfamethoxazole	Outpatient, finished course of antibiotics
48-yr-old male with HIV/AIDS, chronic hepatitis B, found unresponsive with cardiac arrest found to have a brain mass in the basal ganglia	None	IgM: negative (6 days) IgG: positive (1 day)	None	*Toxoplasma gondii* (392) (5 days)	No, already on empiric trimethoprim-sulfamethoxazole	Discharged to hospice with sulfadiazine, clindamycin, and corticosteroid taper
39-yr-old male with HIV/AIDS presenting with encephalopathy and multiple thalamic and brainstem lesions	None	None	None	*Toxoplasma gondii*: not quantified Pseudomonas aeruginosa: not quantified (3 days)	No, already on empiric trimethoprim-sulfamethoxazole	Discharged home with trimethoprim-sulfamethoxazole

*Note*: Co-detection of additional organisms by mcfDNA NGS occurred in four of five patients.

MPM values were recorded when reported; “not quantified” indicates the organism was detected but no quantitative MPM value was provided on the clinical report. Abbreviations: ART, antiretroviral therapy; CSF, cerebrospinal fluid; D+/R−, donor seropositive/recipient seronegative; LTACH, long-term acute-care hospital; mcfDNA NGS, microbial cell-free DNA next-generation sequencing; MPM, molecules per microliter; PCR, polymerase chain reaction; TAT, turnaround time (order to result); TMP-SMX, trimethoprim-sulfamethoxazole.

Plasma mcfDNA NGS detected *T. gondii* in all five cases. Median TAT for mcfDNA NGS was 3 days (range 2–5), compared with 2 days (range 1–2) for Toxoplasma IgG and 5.5 days (range 3–9) for IgM. Two patients underwent CSF *T. gondii* PCR testing (TAT, 3 and 7 days), no patient underwent blood *T. gondii* PCR, and one patient had diagnostic confirmation by brain biopsy.

In two cases, mcfDNA NGS directly prompted anti-Toxoplasma therapy (one transplant recipient with neurologic symptoms and one outpatient with systemic illness). In three cases with compatible CNS imaging, mcfDNA NGS supported ongoing empiric therapy. Co-detection of additional organisms occurred in four of five patients.

## Discussion

In this five-patient case series, plasma mcfDNA NGS returned with a median TAT of 3 days and, in our institutional workflow, preceded send-out IgM and CSF PCR testing when obtained. In two patients, the result initiated targeted anti-Toxoplasma therapy, including a donor-seropositive/recipient-seronegative heart transplant recipient with neurologic symptoms but non-diagnostic imaging and an outpatient with prolonged febrile hepatitis. In patients with classic ring-enhancing lesions, plasma mcfDNA supported continued empiric therapy while confirmatory studies were pending.

Our findings complement prior reports suggesting that plasma mcfDNA sequencing can detect both CNS and non-CNS toxoplasmosis. Roy et al described toxoplasma pneumonitis in AIDS diagnosed by plasma NGS before later recognition of CNS lesions.^8^ Goren et al identified eight cases of *T. gondii* infection by plasma cfDNA sequencing in immunocompromised hosts, including clinically unsuspected and non-CNS presentations.^9^

Plasma mcfDNA may be most useful when conventional diagnostics are equivocal or infeasible. In advanced HIV, toxoplasmosis is often diagnosed presumptively based on compatible imaging and clinical response, with brain biopsy generally reserved for non-responders after 10–14 days.^2^ CSF PCR is highly specific but variably sensitive, with highest yield before or within the first week of therapy.^2,4^ In our series, mcfDNA NGS often returned before send-out IgM or CSF PCR and provided actionable confirmation without lumbar puncture or brain biopsy.

Transplant recipients represent a distinct diagnostic context. Donor-derived toxoplasmosis is strongly associated with donor-seropositive/recipient-seronegative mismatch, particularly after heart transplantation, and disease can occur before seroconversion, limiting the utility of serology in early infection.^3^ The transplant case in our cohort illustrates this challenge: IgG remained negative, and IgM returned later, whereas plasma mcfDNA identified *T. gondii* within 2 days and prompted initiation of therapy. No patient underwent blood *T. gondii* PCR. In allogeneic hematopoietic cell transplantation, serial blood qPCR has been proposed for preemptive early detection, although blood PCR can remain negative in established disease.^10^

The open-ended nature of plasma mcfDNA testing necessitates diagnostic stewardship. Co-detection of additional taxa was common in our patients and is widely reported in clinical practice, where interpretation depends on pretest probability and syndrome compatibility.^6,7^ In our series, additional detections did not meaningfully change management because the T. gondii result was interpreted alongside host factors, imaging, and conventional testing rather than as a stand-alone diagnosis. A positive plasma mcfDNA result should therefore not be considered diagnostic in isolation, and false-positive or clinically irrelevant detections remain possible, particularly when orthogonal confirmation is unavailable or multiple organisms are reported. This contextual interpretation may be more difficult outside infectious diseases-supported or multidisciplinary care.

Plasma mcfDNA also has inherent limitations: it cannot localize infection, quantitative values (MPM) have not been validated as a marker of toxoplasmosis disease burden, and false-negative results can occur, particularly in isolated CNS disease or after therapy. A negative plasma mcfDNA result should therefore not dissuade empiric treatment when clinical suspicion is high. Our study is limited by retrospective design, small sample size, possible false-positive attribution in unconfirmed cases, selection bias toward patients in whom clinicians already pursued plasma mcfDNA testing, and TAT comparisons that reflect local laboratory workflows. Prospective multicenter studies are needed to define optimal patient selection and timing and to evaluate whether plasma mcfDNA meaningfully reduces invasive procedures, antimicrobial exposure, or time to appropriate therapy.

## Data Availability

De-identified data supporting the findings of this study are available from the corresponding author upon reasonable request.

## References

[1] Elsheikha HM , Marra CM , Zhu XQ. Epidemiology, pathophysiology, diagnosis, and management of cerebral toxoplasmosis. Clin Microbiol Rev 2020;34:e00115–e00119. doi:10.1128/CMR.00115-19. PMC7690944 33239310

[2] Vidal JE. HIV-related cerebral toxoplasmosis revisited: current concepts and controversies of an old disease. J Int Assoc Provid AIDS Care 2019;18:2325958219867315. doi:10.1177/2325958219867315. 31429353 PMC6900575

[3] Derouin F , Pelloux H. ESCMID study group on clinical parasitology. Prevention of toxoplasmosis in transplant patients. Clin Microbiol Infect 2008;14(12):1089–1101. doi:10.1111/j.1469-0691.2008.02091.x. 19018809

[4] Cingolani A , De Luca A , Ammassari A , et al. PCR detection of *Toxoplasma gondii* DNA in CSF for the differential diagnosis of AIDS-related focal brain lesions. J Med Microbiol 1996;45:472–476. doi:10.1099/00222615-45-6-472. 8958252

[5] Blauwkamp TA , Thair S , Rosen MJ , et al. Analytical and clinical validation of a microbial cell-free DNA sequencing test for infectious disease. Nat Microbiol 2019;4:663–674. doi:10.1038/s41564-018-0349-6. 30742071

[6] Hogan CA , Yang S , Garner OB , et al. Clinical impact of metagenomic next-generation sequencing of plasma cell-free DNA for the diagnosis of infectious diseases: a multicenter retrospective cohort study. Clin Infect Dis 2021;72:239–245. doi:10.1093/cid/ciaa035. 31942944

[7] Park SY , Chang EJ , Ledeboer N , et al. Plasma microbial cell-free DNA sequencing from over 15,000 patients identified a broad spectrum of pathogens. J Clin Microbiol 2023;61:e01855–e01822. doi: 10.1128/jcm.01855-22. 37439686 PMC10446866

[8] Roy M , Siddique N , Bathina B , Ahmad S. Rare presentation of toxoplasma pneumonitis in the absence of neurological symptoms in an AIDS patient and use of next-generation sequencing for diagnosis. Eur J Case Rep Intern Med. 2020;7:001862. doi:10.12890/2020_001862. 33194865 PMC7655009

[9] Goren LR , Lehman AC , Drozdov D , Thielen BK. Plasma metagenomic cell-free DNA sequencing identifies clinically unsuspected cases of *Toxoplasma gondii* infection in immunocompromised patients. Access Microbiol 2025; doi: 10.1099/acmi.0.001068.v1.

[10] Aerts R , Mehra V , Groll AH , European Conference on Infections in Leukaemia group et al. Guidelines for the management of *Toxoplasma gondii* infection and disease in patients with haematological malignancies and after haematopoietic stem-cell transplantation: guidelines from the 9th European Conference on Infections in Leukaemia, 2022: Lancet Infect Dis 2024;24:e291–e306. doi:10.1016/S1473-3099(23)00495-4. 38134949

